# Silicon Based Coplanar Capacitive Device for Liquid Sensor Applications

**DOI:** 10.3390/s21175958

**Published:** 2021-09-05

**Authors:** Andrea G. Martinez-Lopez, David E. Guzmán-Caballero, Israel Mejia, Julio C. Tinoco

**Affiliations:** 1 Micro and Nanotechnology Research Centre (MICRONA), Universidad Veracruzana, Veracruz 94294, Mexico; andmartinez@uv.mx; 2 Facultad de Ingeniería de la Construcción y el Hábitat (FICH), Universidad Veracruzana, Veracruz 94294, Mexico; 3 Centro de Ingeniería y Desarrollo Industrial (CIDESI), Querétaro 76125, Mexico; david.guzman@cidesi.edu.mx (D.E.G.-C.); israel.mejia@cidesi.edu.mx (I.M.)

**Keywords:** capacitive coplanar sensor, liquid sensor, parasitic capacitances

## Abstract

The development of silicon-based sensor devices has enabled the possibility to pursue novel integrated smart sensor technologies. Under this scenario, capacitive sensor devices are one viable option for implementing different kinds of applications. In this paper, an interdigitated coplanar capacitive device fabricated over a silicon substrate is presented and its potential use as liquid sensor is demonstrated. Additionally, a detailed capacitance model, which includes the parasitic capacitances introduced by the silicon substrate, was developed. The capacitance model has been theoretically validated through finite-element simulations as well as experimentally by comparison with fabricated devices. A polydimethylsiloxane mold has been fabricated and bonded to the sensor device with the aim of defining a cavity to collect the liquid sample into the device’s active region. The active capacitance component correlates to the electric field coupling between adjacent metal lines. Therefore, any change to the dielectric constant of the medium above the coplanar metal lines will produce a change to the device capacitance. Finally, the main guidelines for device performance improvement are depicted.

## 1. Introduction

Recent advances in microfabrication technologies have allowed for the possibility to develop novel sensor devices for a variety of applications such as gas sensors, photodetectors, chemical and biological sensors, as well as liquid sensor devices [1,2,3]. Different kinds of structures and materials have demonstrated that they are suitable for the development of capacitive sensor devices. One of the simplest and most useful structures consist of a parallel-plates capacitor, where the dielectric medium or the geometrical relation between the plates is used in order to produce a change on the device capacitance [4,5,6,7,8,9,10]. Additionally, the use of coplanar capacitive structures (CCS) has demonstrated the possibility to implement sensor devices [11,12,13,14,15]. CCS sensors use the fringing electric field coupling between two adjacent metal lines. Hence, if the medium above the lines changes its electrical properties, the overall sensor capacitance will be modified. However, the presence of the different materials as well as the device geometry promotes the existence of different structures, which usually produces parasitic capacitances. Therefore, an adequate full device analysis, including the parasitic elements, is of importance to the purpose of properly determining the device performance.

Different materials and configurations have been used to develop different kinds of capacitive sensors. For instance, flexible, stretchable, as well as wearable capacitive devices have been proposed for sensor applications [16,17,18]. However, integration with standard digital technology remains an important concern. On the other hand, the fabrication of sensor devices based on silicon technology assures the possibility to easily integrate the sensor with the standard semiconductor technology. This fact affords the opportunity to integrate sensor devices with different electronic circuitry, as well as the benefits of digital processing and storage, giving the possibility to develop functional smart sensor applications. However, the knowledge of the impact of the silicon substrate on the overall device performance is necessary in order to improve the design and device performance.

In this contribution, an interdigitated coplanar capacitive sensor device fabricated on silicon substrate is presented. A detailed capacitance model, which includes the parasitic components, is introduced and validated based on ATLAS simulations, as well as fabricated devices. Finally, the potential application as liquid sensor is demonstrated, and the silicon substrate impact is addressed.

## 2. Coplanar Capacitive Sensor

In this section a description of the fabrication process as well as the physical implementation of the capacitive sensor is addressed.

### 2.1. Fabrication Process

**Figure 1:**
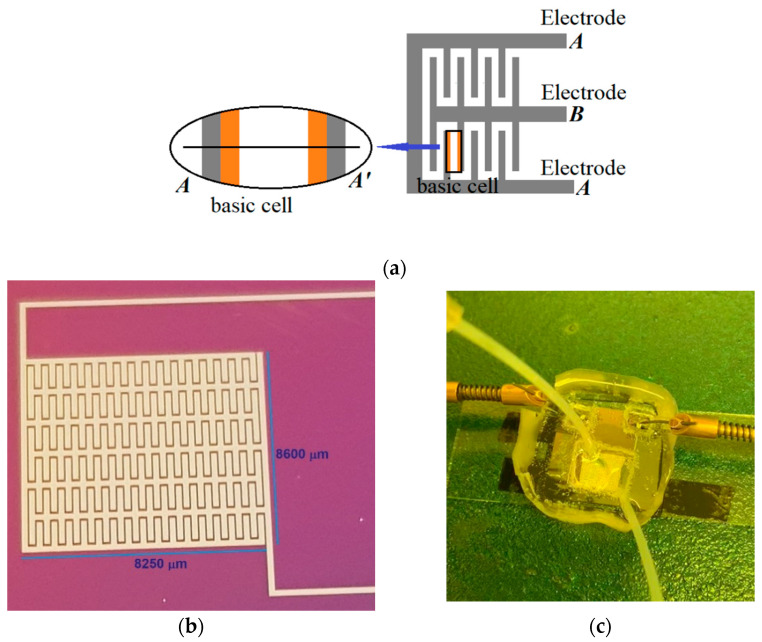
The main characteristics of the coplanar capacitive sensor (CCS) are shown: (**a**) The schematic structure is shown, and the basic cell of the device is defined; (**b**) An image of the fabricated device over a silicon substrate with 200 μm metal width; (**c**) The physical device implantation for liquid sensor applications.

Coplanar capacitive sensors are fabricated using sputtering deposition of aluminum with a thickness of 100 nm. Three different widths: 150, 200, and 250 μm were patterned on a silicon substrate covered by 300 nm SiO_2_. The Al layer was patterned by standard photolithography to define an interdigitated structure with metal lines spacing of 50 μm to define the device’s active region. Figure 1a shows a schematic representation of the device’s active region. Subsequently, by a chemical vapor deposition technique, a 300 nm thin film of parylene-c film was deposited over the structure with the objective of electrically isolating the device’s metal lines from the liquid sample. Figure 1b shows the picture of the fabricated devices

### 2.2. Sensor Physical Implementation

The liquid sensor implementation requires the definition of a microcavity to collect the liquid sample, which includes inlet and outlet feed lines. This cavity was fabricated by molding polydimethylsiloxane (PDMS). The PDMS was prepared by mixing the elastomer and the curing agent (Sylgard 184, Dow Corning), in a ratio 10:1. After molding, a hot-plate process at 80 °C for 40 min was performed. Finally, the mold was bonded to the silicon device by a hot-plate process at 80 °C for 5 min. Figure 1c shows the final device implemented for liquid sensor applications.

## 3. Sensor Model

**Figure 2:**
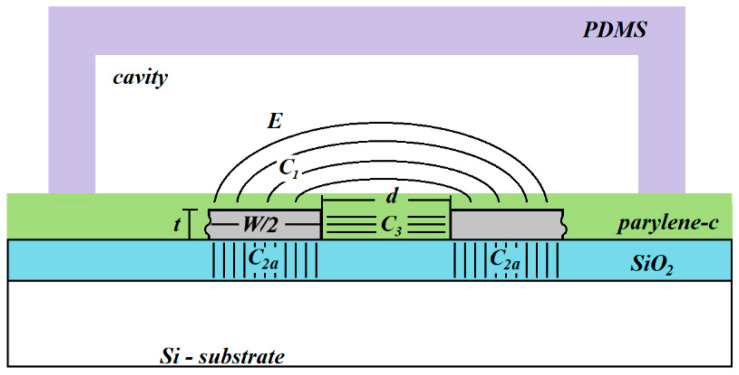
Coplanar capacitive sensor cross section and the main capacitive components. Additionally, the geometrical parameters are shown: *W* is the metal width, *d* is the spacing between the metal lines, and *t* is the metal thickness.

Figure 2 shows the device cross-section along the A-A′ cut of the basic cell indicated in Figure 1a. As can be observed, the sensor is composed of three main capacitive components, which will be described afterwards.

### 3.1. Capacitive Component C_1_

This capacitive component corresponds to the fringing electric field coupling between both metal lines, which is the active device capacitance. As can be observed in Figure 2, the fringing electric field lines pass along two regions, the very thin parylene layer as well as the microcavity. Hence, this component will be defined by an effective dielectric (*k_eff_*) constant defined by the combination of the parylene dielectric constant (*k_par_*) and the dielectric constant of the cavity medium (*k_C_*). When the sensor is empty, the medium into the cavity is air and thus the dielectric constant of the cavity is one (*k_C_* = 1). This scenario is defined as the reference condition. Once a liquid is injected into de sensor, the cavity dielectric constant will change and therefore the effective dielectric constant of the *C*_1_ coupling will be higher than the reference value. Therefore, the overall device capacitance will be increased from its reference value due to the presence of a liquid into the cavity.

Based on previous studies [14,15], the length normalized *C*_1_ component can be defined as:

(1) $$C_{1}=\beta \frac{k_{eff}\varepsilon_{0}}{\pi }\ln\left[1+\frac{W}{d}+\sqrt{{\left(1+\frac{W}{d}\right)}^{\gamma }-\delta }\right]$$

where *β, γ*, and *δ* are fitting parameters.

### 3.2. Capacitive Component C_2_

Due to the conductivity of the silicon substrate, it will act as a virtual electrode, which will promote an electric coupling with each metal line. Therefore, this capacitance will correspond to a series arrangement of two identical components (*C_2a_*), each one defined as a parallel-plate structure formed between the metal line and the virtual electrode. Therefore, the length normalized *C*_2_ component can be defined as:

(2) $$C_{2}=\frac{1}{2}\times \frac{k_{ox}\varepsilon_{0}}{t_{ox}}\times \frac{W}{2},$$

where *k_ox_* and *t_ox_* are, respectively, the SiO_2_ dielectric constant and thickness.

### 3.3. Capacitive Component C_3_

Due to the metal lines thickness, there will be a lateral electric field coupling between both metal lines. This component corresponds to a parallel-plate capacitor where the plate length corresponds to the metal line thickness. Hence, the length normalized capacitance corresponding to this component can be determined as:

(3) $$C_{3}=\frac{k_{par}\varepsilon_{0}}{d}\times t$$

### 3.4. Total Capacitive Sensor Model

As can be seen from Figure 2, the three capacitive components appear in a parallel configuration. Thus, the total device capacitance (*C_T_*), considering that the basic cell corresponds to the half of the structure, is defined as:

(4) $$C_{T}=2L_{eff}\left(C_{1}+C_{2}+C_{3}\right),$$

where *L_eff_* = *α* × *L* corresponds to the effective device length, *α* is a fitting parameter and *L* corresponds to the device length.

## 4. Simulation Results

**Figure 3:**
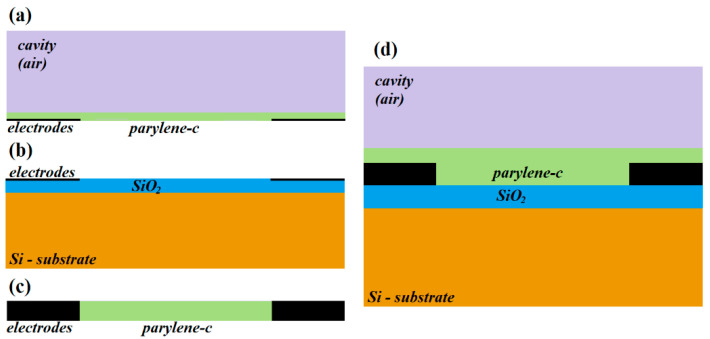
Schematic representation of the set of structures used for simulation routines. Structures shown in (**a**–**d**) are used for simulating the capacitance components *C*_1_, *C*_2_, *C*_3_ as well as *C_T_*, respectively.

The sensor behavior has been validated by ATLAS simulation. Different sets of simulations have been performed with the aim of verifying the main capacitive components. Finally, a set of simulations of the full structure were executed to verify the overall device behavior. Figure 3 summarizes the structures utilized for simulation processes.

**Figure 4:**
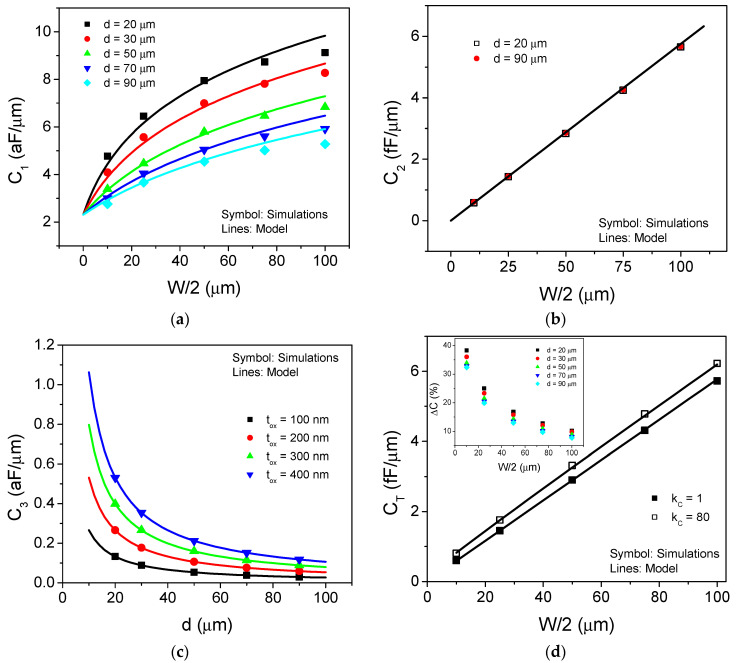
Comparison between simulated and modelled results for: (**a**) Component *C*_1_ vs. the metal line width for several values of lines spacing. The fitting parameters are defined as: *β* = 1.25; *γ* = 1; *δ* = 0.25; (**b**) Component *C*_2_ vs. the metal line width, the separation of the metal lines was 20 and 90 μm, respectively; (**c**) Capacitive component *C_3_* vs. the metal line spacing for several values of metal line thickness; and (**d**) Total capacitance *C_T_* vs. the metal line width, considering two different mediums into the cavity and metal lines spacing of 50 μm. In the inset, the capacitance change for the simulated devices is shown.

The *C*_1_ component was simulated using the structure shown in Figure 3a. Air is considered the medium in the cavity, and Figure 4a shows the comparison between the simulated and modelled capacitance vs. the metal line width for several values of *d*. As can be seen, the capacitance increases as the width of the metal line does. On the other hand, if the spacing between the metal lines is wider, the electric coupling is diminished, and the capacitance consequently decreases.

The *C*_2_ component was simulated according to the structure shown in Figure 3b. Additionally, Figure 4b shows the comparison between the simulated and modelled results of capacitance, *C*_2_ vs. metal line width. As can be observed, *C*_2_ exhibits a linear dependence with the metal width, and it is independent from the spacing between the metal lines as expected from Equation (2). Additionally, the *C_3_* component was simulated using the structure shown in Figure 3c. Figure 4c shows the comparison between the simulated and modelled results vs. the spacing between the metal lines at several thickness values. Additionally, due to the very thin metal lines, the *C_3_* component becomes negligible.

Additionally, the full device has been simulated using the structure shown in Figure 3d, where two different conditions are considered: (i) the reference one, which corresponds to an empty sensor with air in the cavity medium; and (ii) considering the presence of certain liquid into the cavity, and for this purpose the region above the parylene region is defined with a dielectric constant of 80 with the aim of considering that the cavity is filled with water. Figure 4d shows the comparison between simulated and modeled device capacitance vs. the metal width for both mediums into the cavity and 50 μm for metal line spacing. In the inset, the capacitance changes (*ΔC*) for the different metal spacing values are shown. As can be observed, *C_T_* exhibits a linear dependence on the metal line width, confirming that the dominant capacitance of the whole device is the parallel-plate component *C*_2_ as Figure 4a–c shows.

Furthermore, the fact that any change to the dielectric constant of the medium above the parylene layer will produce a change to the *C*_1_ component, and as a consequence on the overall device capacitance, is verified. Additionally, as the metal width is reduced, the capacitance change is more significant. It is noteworthy that both *C*_1_ and *C*_2_ components show a reduction in the metal width, however the *C*_2_ reduction is more important, hence *ΔC* is improved. These results clearly show that silicon substrate produces an important impact on the device performance. Therefore, device improvement strategies must consider both the *C*_2_ reduction as well the increment of *C*_1_. According to Equations (1) and (2), an increase in the SiO_2_ film thickness will produce a reduction in the *C*_2_ component, while a reduction in the metal line spacing will cause an increase in *C*_1_. Moreover, a reduction in the metal width could imply a trade-off on the device performance. Additionally, some strategies with the aim to electrically decouple the silicon substrate from the total capacitance can be considered for device optimization.

## 5. Experimental Results

**Figure 5:**
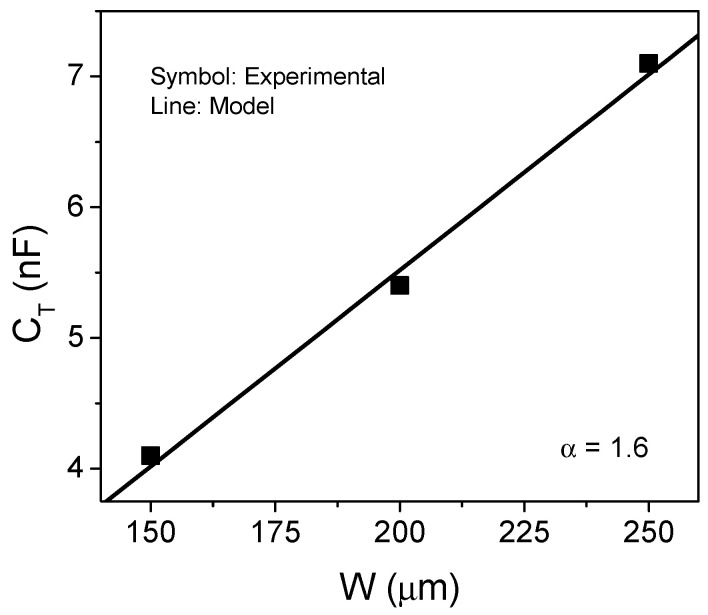
Comparison between measured and simulated capacitance at reference condition for the different fabricated devices. An effective length of 52 cm is obtained.

The sensors fabricated with different metal line widths were measured under the reference condition, i.e., with air in the cavity, and compared with the model defined by Equation (4). Figure 5 shows the comparison between the measured and the modelled capacitance vs. the metal width. As can be seen, the linear dependence observed in the simulations is experimentally confirmed. The devices have a length of about 16.2 cm and a fitting parameter (*α*) with a value of 1.6 was determined. Therefore, a sensor effective length of about 52 cm was achieved

**Figure 6:**
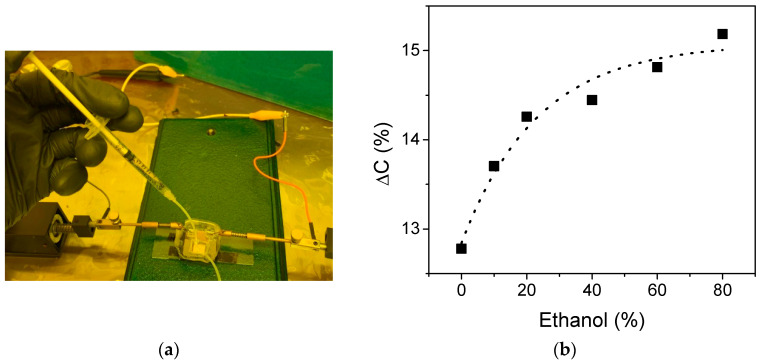
Implementation of the CSC as a liquid sensor device. (**a**) Physical implementation set-up. (**b**) Physical measurement of the capacitance change (Δ*C*) vs. ethanol concentration aqueous solution.

Afterwards, a device with 200 μm metal line width was implemented as a liquid sensor, as Figure 1c shows. Finally, an aqueous solution with ethanol at different concentrations was injected into the device as Figure 6a shows and the capacitance change was measured. Figure 6b shows the capacitance change vs. the ethanol concentration. As can be seen, the implemented liquid sensor exhibits a capacitance change of about 12.7% in the presence of water, which is consistent with the simulation results shown in the inset of Figure 4d. Additionally, in the presence of ethanol solution the capacitance change is slightly increased until 15% as the alcohol concentration is increased to 80%. These results experimentally confirm the possibility of implementing a liquid sensor in a silicon substrate.

## 6. Conclusions

A capacitive coplanar sensor device fabricated on silicon substrate has been implemented for liquid sensor applications. A PDMS microcavity has been designed and bonded to the silicon-based device with the aim of properly delivering the sample steam to the sensor’s active area. Additionally, the main sensor capacitance components have been analyzed and a sensor model has been developed. Furthermore, the impact of the silicon substrate due to a parasitic capacitance component has been presented. Finally, experimental sensor behavior has been demonstrated using an ethanol aqueous solution and it is shown that the total device capacitance is increased from 12.7% for deionized water content to 15% when 80% ethanol content is used.

## Data Availability

Not applicable.
